# Positioning the Red Deer (*Cervus elaphus*) Hunted by the Tyrolean Iceman into a Mitochondrial DNA Phylogeny

**DOI:** 10.1371/journal.pone.0100136

**Published:** 2014-07-02

**Authors:** Cristina Olivieri, Isolina Marota, Ermanno Rizzi, Luca Ermini, Letizia Fusco, Alessandro Pietrelli, Gianluca De Bellis, Franco Rollo, Stefania Luciani

**Affiliations:** 1 Laboratory of Molecular Archaeo-Anthropology/ancient DNA, School of Biosciences and Veterinary Medicine, University of Camerino, Camerino, Italy; 2 Institute of Biomedical Technologies, National Research Council, Segrate, Italy; 3 Centre for GeoGenetics, Natural History Museum of Denmark, University of Copenhagen, Copenhagen, Denmark; University of Perugia, Italy

## Abstract

In the last years several phylogeographic studies of both extant and extinct red deer populations have been conducted. Three distinct mitochondrial lineages (western, eastern and North-African/Sardinian) have been identified reflecting different glacial refugia and postglacial recolonisation processes. However, little is known about the genetics of the Alpine populations and no mitochondrial DNA sequences from Alpine archaeological specimens are available. Here we provide the first mitochondrial sequences of an Alpine Copper Age *Cervus elaphus*. DNA was extracted from hair shafts which were part of the remains of the clothes of the glacier mummy known as the Tyrolean Iceman or Ötzi (5,350–5,100 years before present). A 2,297 base pairs long fragment was sequenced using a mixed sequencing procedure based on PCR amplifications and 454 sequencing of pooled amplification products. We analyzed the phylogenetic relationships of the Alpine Copper Age red deer's haplotype with haplotypes of modern and ancient European red deer. The phylogenetic analyses showed that the haplotype of the Alpine Copper Age red deer falls within the western European mitochondrial lineage in contrast with the current populations from the Italian Alps belonging to the eastern lineage. We also discussed the phylogenetic relationships of the Alpine Copper Age red deer with the populations from Mesola Wood (northern Italy) and Sardinia.

## Introduction

One of the most remarkable features of the glacier mummy known as the Tyrolean Iceman (also called Similaun Man or Ötzi) is the exceptional quality of preservation of clothes and equipment found with her at the time of the discovery. According to the radiocarbon dating the finding dates back to approximately 3,300 to 3,200 years BC., corresponding to the Copper Age. Thus, the find offers a unique opportunity to deeply examine the clothing of a Neolithic man who lived somewhere south of the Alpine mountains. Over the years numerous scientific investigations have been performed on the garment to understand the subsistence strategies based on the plant and animal resource utilization. Early studies were conducted in the 1990s and were designed to molecular analyze samples of grass most likely coming from the shoes and the cape of Ötzi [1], [2]. Later, studies carried out through matrix-assisted laser desorption/ionization time-of-flight mass spectrometric (MALDI-TOF MS) method and microscopy analyzed fur specimens from the accoutrement and detected chamois, brown bear, goat, cattle, canid species, sheep and red deer [3]–[5]. In 2012 Olivieri et al., [6] identified and sequenced mitochondrial DNA (mtDNA) fragments of sheep (*Ovis aries*) from black animal hair shafts recovered from Ötzi's clothes. The study described the oldest mitochondrial sequences from European sheep known until then and a sheep's lineage that has not yet been identified in modern sheep populations.

During Neolithic red deer (*Cervus elaphus*) was among wild animals, a valuable resource for clothing, food and tools; the antlers, in particular, played an important part for the manufacture of tools and weapons. The analysis of the Ötzi's equipment revealed the use of this material for making the retoucher (a tool for working flints) and the tips found in the quiver.

Moreover the DNA reconstruction of the Ötzi's last meal demonstrated the use of red deer meat as a food supply [7].

Today red deer has a large distribution extending from Europe and North Africa through central Asia, and North America. The climatic oscillations occurred in the Quaternary period and, more recently, the human impact through habitat fragmentation, re-introductions or translocations for hunting purposes have heavily altered the red deer populations' genetics [8], [9].

In the last years several phylogeographic studies on red deer have been conducted.

In 2004, Ludt et al., [10] examined the DNA sequence variation of the mitochondrial cytochrome b gene (*cytb*) of 51 populations of *Cervus elaphus* from the entire distribution area of *Cervinae* focusing on Europe and Asia. They found two distinct groups of red deer: a “Western Red Deer” clade consisting of four sub-clade (Western-Europe, Balkan, Middle East and Africa) and an “Eastern Red Deer” clade consisting of three sub-clade (North-Asia/America, South-Asia and East-Asia).

Skog et al., [11], analyzing the variation at two mitochondrial markers (*cytb* and control region) of European red deer sampled from 39 different locations, identified three main mitochondrial DNA lineages, called haplogroups A, B, and C. Each haplogroup showed a distinct geographical distribution: haplogroup A was mainly located along a south-north axis in Western Europe; haplogroup B, was found only in Sardinia and in Africa and haplogroup C was distributed in Eastern and Central Europe. This distributional pattern has been interpreted as the result of isolation in different refugia during the last glaciation. The western and eastern European lineages (haplogroups A and C) therefore could be associated to an Iberian and Balkan refugium, respectively, while the haplogroup B lineage might have originated from a Sardinian or African refugium.

To investigate the genetic diversity of red deer in Norway Rosvold et al., [12] analyzed mitochondrial DNA (mtDNA) sequences from contemporary and ancient specimens of red deer spanning about 7000 years. Current genetic diversity of Norwegian red deer seems to be a consequence of a postglacial immigration event and a subsequent gradual loss of genetic diversity due to anthropogenic activities. Carden et al., [13] studied the history and the origins of the Irish red deer employing ancient (30,000–1,700 years BP) and modern red deer mtDNA sequences. Eighteen haplotypes were found (IRE1–IRE18) of which six (IRE1–IRE2; IRE15–IRE18) were detected in both contemporary and ancient individuals.

More recently, researchers sampled modern and ancient red deer specimens from the entire European range of the species over the last *c.* 40,000 years [14]. Results described two main European red deer groups: West/central Europe vs South-East Europe and Western Asia arose by red deer expansions from Iberia and Balkans refugia (and possibly from Italy as well) in the last post-glacial warming. However, the role of Italy as a possible refugium could be not clearly inferred as the ancient samples from Italy did not yield DNA.

At the present, few mitochondrial DNA sequences of red deer from Alpine region have been analyzed and no ancient mitochondrial sequences of red deer from this area are available.

In this study we report the first analysis of ancient mitochondrial DNA sequences obtained from a red deer belonging to an Alpine Copper Age population. We targeted a portion of 2,297 base pairs of the mitochondrial DNA encompassing the whole cytochrome b gene, a portion of the tRNA^Thr^ gene, the whole tRNA^Pro^ gene, the complete control region, the whole tRNA^Phe^ gene and a portion of the 12S rRNA gene. We obtained DNA from hair shafts collected from the fur worn by the Tyrolean Iceman. We generated sequences by massive sequencing of pooled amplification products by 454/Roche genome Sequencer and inferred the phylogeny of the Alpine Copper Age red deer within both contemporary and ancient red deer populations.

## Materials and Methods

### Sample

The sample analyzed in this study (No. 324) was retrieved during the second archaeological excavation at the Iceman's discovery site carried out from 4–24 August 1992.

This sample consists of fragments of human skin and animal hairs, and was collected on 21.08.1992, little eastwards of the stone on which the mummy was lying [15].

Currently, the sample is part of the collection held at the South Tyrol Museum of Archaeology. Small subsamples (100–200 mg) were provided to one of us (FR) by Lorenzo Dal Ri, at the time director of the Archaeological Superintendence of Bolzano. No further authorization was required. Once the samples arrived in our laboratory, we performed a preliminary examination of the hair shafts by naked eye and selected the hairs on the base of the colour, character (soft or bristly) and thickness. Colour ranged from black to yellowish brown and to white/grey. To determine the species to which the hairs belonged, we performed the DNA analysis. The genetic analysis of the black animal hair shafts allowed us to identify sheep (*Ovis aries*) [6]. In this study we report the results of the genetic analyses on white/grey hairs.

### Ancient DNA work

All manipulations of ancient DNA were performed at the Laboratorio di Archeo-Antropologia Molecolare/DNA Antico of the Camerino University, devoted exclusively to ancient DNA analysis. All analytical steps prior to DNA amplification were conducted in a room where the operator wears a fullbody sterile suit, gloves, a face screen, and breathing mask (pre-PCR area). The room is equipped with UV lights and a positive-pressure air-filtering system providing 99.97% particle elimination and a complete change of air every 10 min. Amplifications were carried out in a PCR laboratory UV irradiated before and after use. All surfaces in the laboratories are regularly cleaned with bleach.

In the Laboratorio di Archeo-Antropologia Molecolare/DNA Antico no modern both human or animal DNA has ever been introduced.

### DNA extraction

40 mg of hair shafts were cut into small (∼0.5 cm) fragments and manually washed several times with sterile H_2_O to remove any mud, and debris from the outside of the hairs. Digestion was performed overnight at 55°C with rotation, using 600 µl of following digestion buffer: 10 mM Tris-HCl (pH 8.00), 10 mM NaCl, 2% w/v SDS, 5 mM CaCl _2_, 2.5 mM EDTA (pH 8.00), 40 mM dithiothreitol (DTT; Cleland's reagent) and 10% proteinase K solution (>600 Mau/ml, Qiagen). After digestion the resulting liquid solution was used to extract DNA throughout a phenol-chloroform protocol. The DNA fraction was precipitated from the final supernatant by centrifugation at 13,500 g for 5 min after the addition of 1/10 volume of 2 M sodium acetate and 2.5 volumes of cold (−20°C) ethanol. Finally, the DNA precipitates were re-suspended in 20 µl of sterile distilled water and stored at −80°C until use. We prepared extraction blanks throughout the procedure.

### PCR amplification and sequencing

Three regions of the mtDNA genome were targeted and sequenced. The first region, corresponding to a fragment of the 12S ribosomal RNA (rRNA) gene (12S rRNA), was generated using PCR system for mammal DNA (MbosL1269/MbosH1346) amplifying a fragment of 117 bp in length (calculated on the basis of the *Bos taurus* sequence) [7]. The second region corresponds to a 1,140 bp fragment of the *Cervus elaphus* cytochrome b (reference sequence NC007704 positions 14,162–15,301). The third region matches with a 1,157 bp fragment spanning part of the *tRNA^Thr^*, the complete length of tRNA*^Pro^*, the complete mitochondrial control region (CR), the complete length of *tRNA^Phe^*, and a portion of the *12S rRNA* (NC007704 positions 15,308–16,357 and 1–109). In total we sequenced a 2,297 bp long fragment. In order to amplify the second and third region we designed two sets of overlapping primers specific for *Cervus elaphus*, able to cover portions of the mtDNA sequence ranging from 91 bp to 224 bp. The PCR systems were directly tested on the ancient-DNA template, and no positive (i.e., modern DNA) control was used, to minimize the risk of contamination. The list of oligonucleotide primer-pairs utilized and the corresponding annealing temperatures are given in Table S1.

Amplifications were performed in 100 µl of reaction mix of the following composition: 67 mM Tris-HCl (pH 8.8), 16.6 mM (NH4)_2_SO_4_, 0.01% Tween 20, 2.5 mM MgCl_2_, 2.5 U of Taq polymerase (Hot Rescue DNA Polymerase, Diatheva), 200 mM each dNTP, 300 ng each primer, and 1 µl of 1∶40 diluted DNA template. We pre-treated the reaction mixture with DNase (2 enzyme units for 30 min at room temperature) to eliminate any contaminant DNA and subsequently inactivated the DNase by heating to 95°C for 15 min. The thermal profile was as follows: 1 min at 95°C, 50 s at the relevant annealing temperature, and 1 min at 72°C, with a final extension of 10 min at 72°C. The number of cycles ranged from 45 to 55. Amplification products were purified using the High Pure PCR Product purification kit (Roche Molecular Biochemicals, Mannheim, Germany). The product of the Mbos L1269/Mbos H1346 amplification system was cloned using the pGEM-T Easy Vector System (Promega Corp., Madison, WI) and 10 clones were sequenced. The other PCR products were directly sequenced by traditional Sanger sequencing using ABI-Prism 310 automated DNA sequencer (BMR-Genomics sequencing service; University of Padua). The sequences were checked and analyzed by Sequence Scanner version 1.0 (Applied Biosystems, Foster City, CA), and aligned with the program BioEdit v.7.0.9. [16].

### 454 sample preparation and 454 platform sequencing

The purified amplicons pool was quantitated by spectrophotometric measure (NanoDrop ND-1000, Thermoscientific) and analyzed by Agilent Bioanalyzer. About 320 ng of sample were converted in double stranded and MID (multi-Identifier) labelled library following the Roche/454 Rapid Library procedure. The obtained library was quantitated by Real Time PCR (qPCR) using KAPA Library Quant Kits (KAPA Biosystems, MA, USA) and emulsion PCR was performed following the Roche/454 emPCR procedure. After emPCR, the obtained beads were enriched, counted and pyrosequenced onto two lanes of Roche/454 Pico TiterPlate (PTP) subdivided in 4 lanes.

Raw reads (n = 98,272) containing the Roche MID (MID n°8) and primer sequences, were demultiplexed and filtered using an in-house trimming script based on BLAST and the resulting reads (n = 77,440) were mapped against the reference sequence (*Cervus elaphus* mtDNA sequence, NC007704) using the software CLC Genomics Workbench v5.0.1 configured with the following mapping parameters: mismatch cost = 3; insertion/deletion cost = 3; min; length fraction = 0,6; min. similarity fraction = 0,8. The variant call was performed by CLC Genomics Workbench software to generate the report of the called nucleotide at each position for the identification of the phylogenetic variants and miscoding lesions such as the C→T and G→A transitions.

### Nucleotide misincorporation analysis

The consensus sequence for each group of clones, produced by 454/Roche Genome Sequencer, was determined from the shared bases and the remaining interclone base differences (nucleotide misincorporation) were attributed to either post-mortem damage (miscoding lesions) or polymerase errors. The number of type 1 and type 2 transitions were assessed according to Gilbert et al., [17].

In detail, for each group of clones we measured the absolute number of each of the 12 possible base changes (A→C, A→G, A→T, C→A, C→G, C→T, G→A, G→C, G→T, T→A, T→C, T→G) which, due to the complementary nature of DNA, were grouped into six complementary pairings (A→G/T→C, A→T/T→A, A→C/T→G, C→T/G→A, C→G/G→C, C→A/G→T). All the nucleotide misincorporation values were scaled to compensate the mitochondrial fragment nucleotide composition bias. In particular, when the nucleotide misincorporations originated from an underrepresented C and G nucleotide, their values were multiplied by the ratio A+T/G+C in the specific fragment. On the other hand, when the nucleotide misincorporations originated from an underrepresented A and T nucleotide, their values were multiplied by the ratio G+C/A+T in the specific fragment. Insertions and deletions were excluded from the analysis.

### Phylogenetic analyses

In each phylogenetic analysis we adapted the lengths of the alignments to incorporate the maximum number of sequences available in GenBank. This allowed us to compare our data to the published sequences.

Two worldwide *Cervus elaphus* phylogenetic trees were inferred for the mitochondrial regions examined in this study. The first tree employed 38 contemporary sequences to target a fragment of 917 bp of the control region, the second one, was build up on a 1,140 bp fragment of *cytb* with 45 modern sequences (see Table S2, S3 for accession numbers and more detailed informations). The genetic analysis of the Copper Age *Cervus elaphus* within European red deer was carried out employing both phylogenetic trees and phylogenetic networks. The inferred phylogenies were based on 1,118 bp of 17 *cytb* contemporary haplotypes [11] (Table S4) (reference sequence NC007704 positions 14184–15301) and 327 bp of 82 mtCR haplotypes from GenBank consisting of both ancient and modern sequences [11], [12], [13] (Table S5) (reference sequence NC007704 positions 15573–15899).

Sequences were aligned using ClustalW software implemented by the BioEdit program version 7.0.9.

Phylogenetic trees were built up with MEGA5 software [18] employing a maximum likelihood algorithm, while phylogenetic networks were constructed using median joining algorithm [19] as implemented in Network 4.6 (http://www.fluxus-technology.com).

To reduce the probability of incorrect and inaccurate phylogenies in the network and faulty interpretations of global haplogroup relationships, sequence gaps (insertions and deletions in the alignment) were excluded from the analysis except the insertion in position 15581 which in the mitochondrial control region alignment showed to be common to haplotypes belonging to haplogroup C. To achieve a control region easily readable network nucleotide positions were weighted in inverse proportion to the frequency of mutations observed for each position. Nucleotide positions in the *cytb* network instead were equally weighted in all loci.

## Results

### Sample identification and DNA sequencing

To identify the species to which the specimens belong, the DNA extracted was PCR amplified using the MBosL1269/MBosH1346 universal oligonucleotide primer pair for mammal DNA [7].

The PCR product was cloned and 10 clones were analyzed by Sanger sequencing. A BLAST search of the consensus sequence revealed 100% identity to the sequence of *C. elaphus*. The DNA extracted was then PCR amplified using two sets of 13 and 12 oligonucleotide primer pairs covering, respectively, portions of *cytb* and control region. All 25 amplifications gave positive results. To check the specificity of the primer sets, the 25 PCR products were sequenced by conventional Sanger technology before running 454 sequencing. The products were diluted to equal concentrations, pooled and used as a substrate to prepare a library for sequencing using a GS-454/Roche Genome Sequencer (FLX Roche 454 Lifesciences). The sequencer yielded as total of 98,272 reads which after demultiplexing and filtering were reduced to 77,440. A total of 59,971 reads (∼77.44% of the total filtered reads) mapped against the reference genome. The reads were assigned to 25 clonal groups distinct on the base of their position in relation to red deer reference mtDNA sequence (NC007704). The clonal groups were sufficiently covered with a mean of 6,305 reads and a mean nucleotide depth of 2,917X. The deep sequencing data always showed phylogenetic consistency in the subsequent genetic analyses.

The polymorphisms in the Copper Age red deer compared to the NC007704 are reported in Table 1.

**Table 1:** Alpine Copper Age red deer nucleotide polymorphisms relative to reference sequence NC007704.

Nucleotide position	Reference sequence (NC007704)	Alpine Copper Age red deer
108	A	G
14794	C	T
14858	T	C
15372	A	C
15374	T	C
15558	T	A
15678	C	T
15703	A	G
15749	C	T
15791	C	T
15932 ins	-	C
16199 del	T	-
16263	A	G

The consensus sequences were deposited in DNA Data Bank of Japan under accession numbers AB926017 and AB924664 and the raw reads in the NCBI's sequence read archive (SRA) under accession number SRR1259110.

A detailed analysis of the nucleotide misincorporation patterns showed an excess of type 2 transitions over the type 1 (ratio type 2/type 1 ∼2∶1), typical of ancient templates (Table S6).

### Phylogenetic analyses

To investigate the phylogenetic position of the 5,100–5,350 years old *C. elaphus*, we first compared the ancient mtDNA sequences (control region and *cytb*) with the corresponding modern sequences of red deer from the entire distribution area of *Cervinae*.

**Figure 1:**
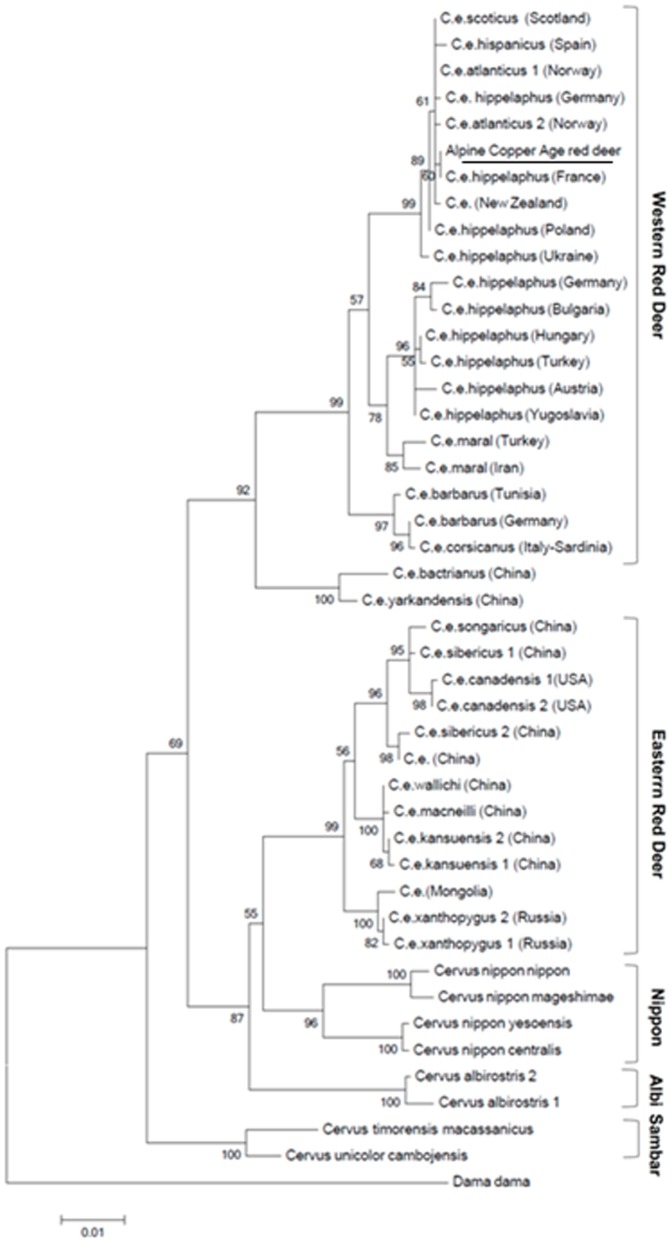
Maximum likelihood tree of the 1,140 bp cytochrome b sequences from worldwide red deer. The evolutionary history was inferred by using the Maximum Likelihood method based on the Tamura-Nei model [20]. The tree is drawn to scale, with branch lengths measured in the number of substitutions per site. Numbers on branches indicate bootstrap support (1,000 replicates).

**Figure 2:**
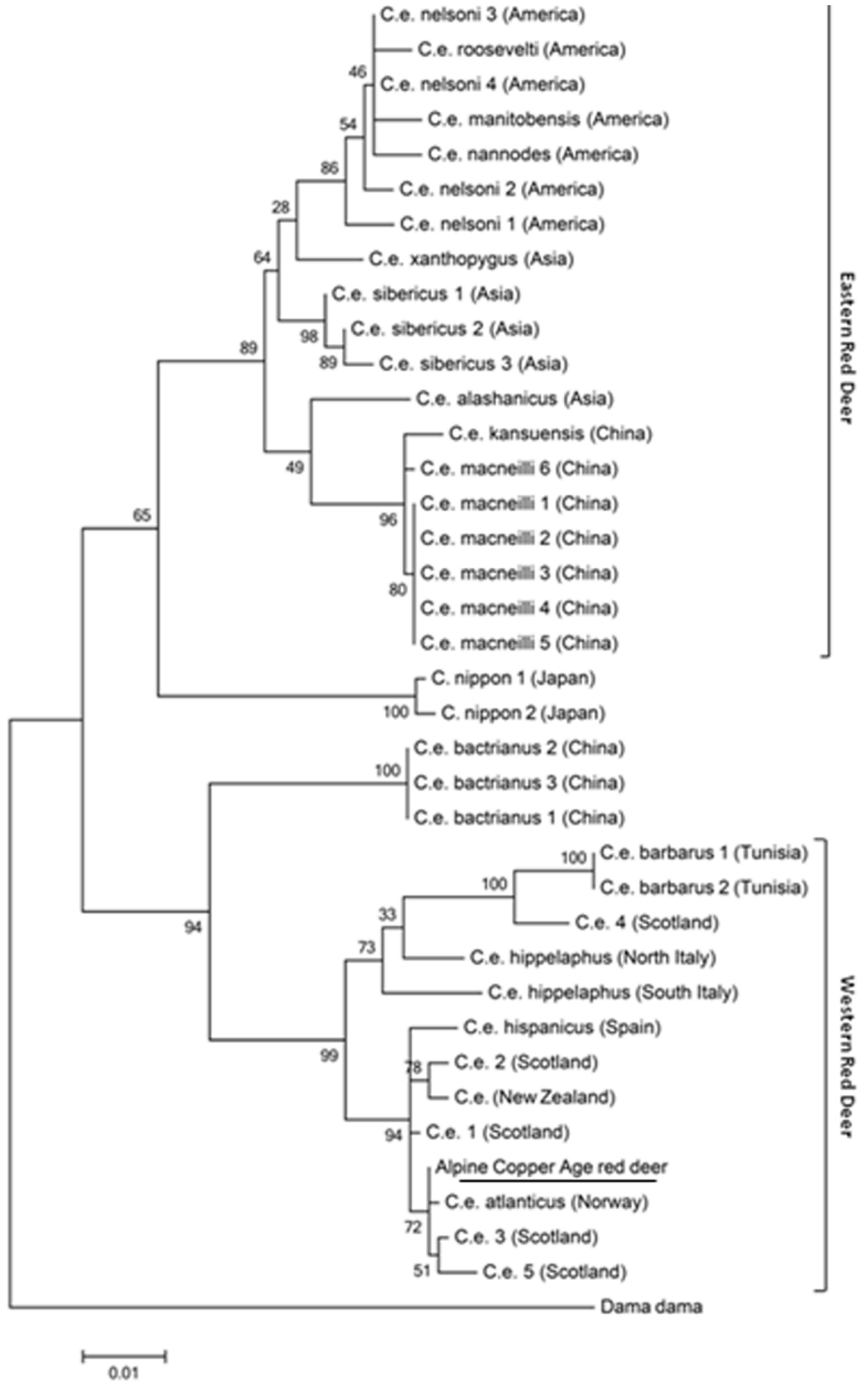
Maximum likelihood tree of the 917 bp mtDNA control region sequences from worldwide red deer. The evolutionary history was inferred by using the Maximum Likelihood method based on the Tamura-Nei model [20]. The tree is drawn to scale, with branch lengths measured in the number of substitutions per site. Numbers on branches indicate bootstrap support (1,000 replicates).

The inferred worldwide maximum likelihood trees for *cytb* and control region are showed in Figures 1 and 2.

Both of these phylogenetic analyses gave similar clustering pattern with the Copper Age red deer's sequence within the Western European subgroup.

We next focused on the European haplotypes and we constructed two phylogenetic networks: one for the *cytb* and one for the control region.

**Figure 3:**
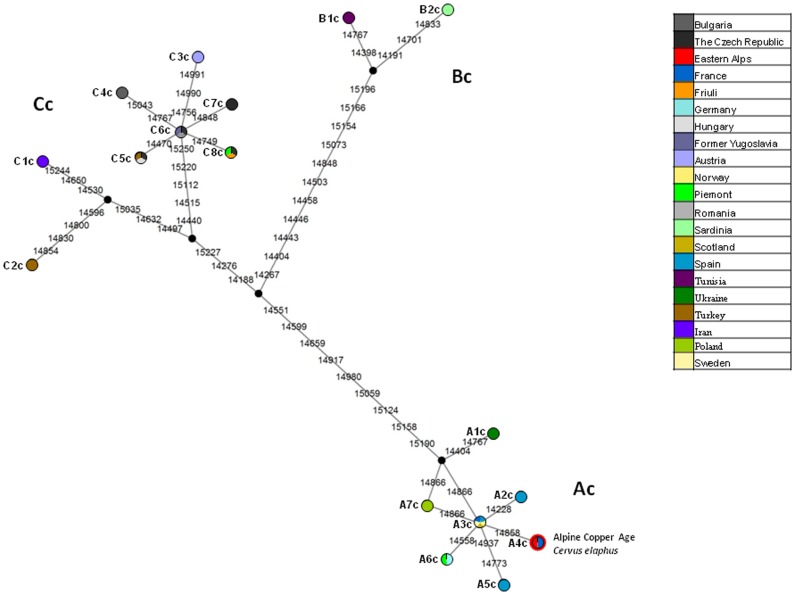
Median-joining phylogenetic network of the 1,118 bp *cytb* sequences depicting the relationship of the Alpine Copper Age red deer with the modern European red deer haplotypes. Each circle refers to one haplotype. Colour of the node indicates the geographic location of the haplotypes. Red colour of circle outline represents the position of the Alpine Copper Age *Cervus elaphus*. Line connecting each circle represents phylogenetic branches. Numbers along each branch are transitions and refer to nucleotide position variants relative to *C. elaphus* reference sequence. Black dots represent median vectors.

The *cytb* network (Figure 3) showed three main branches corresponding to haplogroups Ac, Bc and Cc as already described by Skog et al., [11].

A substantial number of mutations divides each haplogroup but the network lacks of definition as only 17 haplotypes mined from the literature can be included. On the basis of this evidence it was impossible inferring possible loci characterizing evolutionary branches.

In the network the Alpine Copper Age red deer clusters within the haplogroup Ac. In contrast, haplotypes found in modern Alpine samples taken from sites close to the archaeological area where the ancient specimen was found (Austria, Friuli) are located in the haplogroup Cc (C3c and C8c, respectively).

The network also shows that haplotype B2c of *C. e. corsicanus* from Sardinia is genetically very distant from the Alpine Copper Age red deer 's haplotype (A4c) with 25 differences on an alignment of 1,118 bp.

The *cytb* sequence of the Alpine Copper Age red deer was also included in a maximum likelihood tree using the same haplotypes employed in the network. The results showed the ancient sequence placed in the tree in the same position inferred by the network analysis (Figure S1).

**Figure 4:**
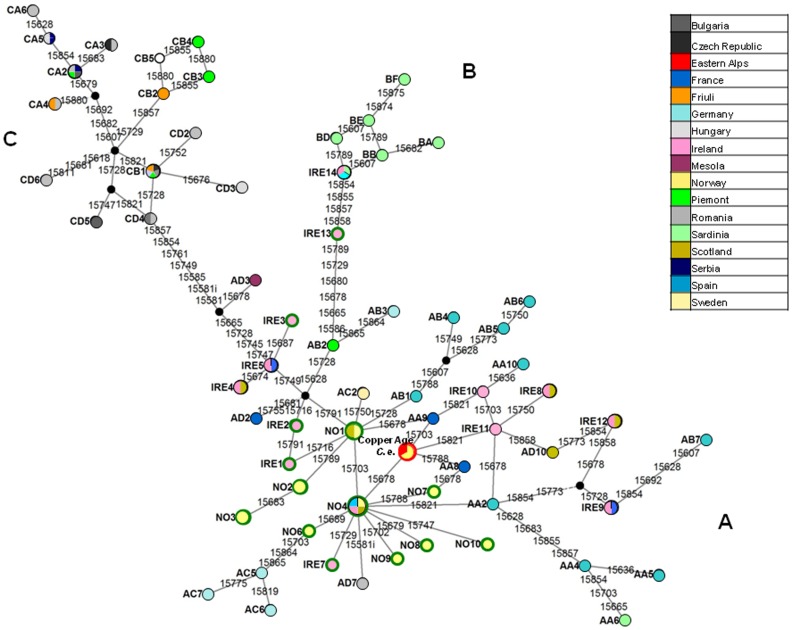
Median-joining phylogenetic network of the 327 bp mtDNA control region sequences depicting the relationship of the Alpine Copper Age red deer with the European (modern and ancient) haplotypes. Each circle refers to one cluster and size corresponds to the number of haplotypes described in the literature. The clusters including more haplotypes have the following composition: NO4 (NO4, AA1, IRE6, AD6); Alpine Copper Age *C.e.* (Alpine Copper Age *C.e.*, AA7, NO5); NO1 (NO1, AD5, AC1); IRE8 (IRE8, AD8); IRE12 (IRE12, AD9); IRE4 (IRE4, AD4); CA2 (CA2, CA1); CB1 (CB1, CD1); IRE5 (IRE5, AD1); IRE9 (IRE9, AA3); IRE14 (IRE14, BC); NO2 (NO2, AC3); NO3 (NO3, AC4). Colours within the nodes represent the geographic location of the haplotypes. The red and green colours of the circle outlines represent the Alpine Copper Age *Cervus elaphus* and ancient samples (Norway and Ireland), respectively. Line connecting each circle represents phylogenetic branches. Numbers along each branch are transitions and refer to nucleotide positions variants relative to *C. elaphus* reference sequence. Black dots represent median vectors.

In order to get more insights on the genetic relationships of the Alpine Copper Age red deer we constructed a network using a reduced alignment of 327 bp of the mtDNA control region to include all European contemporary and ancient sequences stored in the GenBank covering the same portion of the genome (Figure 4).

Like the ancient red deer sequences previously described, the Alpine Copper Age red deer also falls within the haplogroup A in accordance to the *cytb* results. Moreover, the ancient Alpine haplotype exactly matches with haplotypes AA7 and NO5, identified by Skog el al., [11] and Rosvold et al., [12], respectively. Both haplotypes have been found in Norway and NO5 has also been identified in modern samples and ancient samples dating back to c. 2,000–2,500 yr BP and c. 500–1,500 yr BP.

Samples of age more similar to the Alpine Copper Age red deer (5,100–5,350 years old) are located in different haplotypes that differed from our specimen by 1 bp (NO4) or 2 bp (NO1, NO7, NO9, NO10).

Interestingly, the Alpine Copper Age red deer belongs to haplogroup A in contrast with red deer from Alpine regions of Italy that fall within the haplogroup C (most of samples collected from Piedmont and the whole sampling from Friuli) (Table S5).

In the network the haplotype AD3, found exclusively in red deer sampled in the Mesola Wood (Emilia Romagna, northern Italy) is genetically very distant from the Alpine Copper Age and maintains the ambiguous placement between haplogroups A and C in line with previous results [11].

The maximum likelihood tree inferred using the same haplotypes of the network also supports this result (Figure S2).

### Analysis of the median-joining network of mtDNA control region

The network identified 48 positions carrying single nucleotide substitutions where one of which, the position 15581 showed also an insertion (15581i). Of the 82 haplotypes initially included in the newtork, 65 revised haplotypes were detected into the analyzed region, and the frequency of each mutation was also identified. The highest frequency corresponded to positions 15678 and 15854, which may indicate hotspot loci.

The haplogroup A in our network (Figure 4) is affected by a steady reticulate structure impossible to resolve with the dataset employed and therefore mutations characterizing this haplogroup cannot be inferred. Two clusters are detached from haplogroup A as described before [11], [13]. The first of the two is the haploghroup B. The haplogroup B shares two positions 15728 and 15828 with sequence AB2, both loci show a high frequency within the network and thus the connection between haplogroup B and AB2 is not strongly supported. Two loci 15586 and 15680 happen only once in the whole network thus they seem to be strong candidates in defining haplogroup B with the resulting motif: 15586–15680. In addition, considering the position of IRE13 and the latter motif, IRE13 may be regarded as an ancestor of the whole haplogroup B. The four mutations belonging to the branch departing from IRE13 towards haplogroup B happen with a relatively high frequency within the network (the lowest frequency is 3 and belongs to the position 15858) and we do not asses them as strong candidates to define the haplogroup B.

The second cluster identified sequences already recognized as haplogroup C. The node IRE5 (IRE5 and AD1) and the whole haplogroup C share the locus 15749, a relatively high frequency (four times) mutation, which may be the result of a convergent mutation rather than a mutation characterizing the haplogroup C. The long branch departing from the node IRE5 is characterized by different loci with different frequency. The loci 15745, 15761 and 15581, with a frequency of one, are strong candidates in identifying haplogroup C. The locus 15745 is the only one of the three above positions shared with the haplotype AD3 (found in contemporary individuals sampled in Mesola Wood, northern Italy), which a previous publication [11] has shown as of suffering of an uncertain placement within the inferred phylogeny. Here, AD3 branches out at the root of the branch leading to haplogroup C for the mutation in position 15678. Because this is one of the two identified hotspot positions we do not exclude that haplotype AD3 can be thought of as intermediate between A and C.

The position 15581i happens twice in the network bearing each two different bases. In the haplogroup C branch it carries a thymine and in the AD7 haplotype it carries a guanine. Both insertions appear only once in the network and we may incorporate the thymine insertion in the motif characterizing the haplogoup C: 15745,15761, 15581 and 15581.1T.

## Discussion

Fossil records attest the presence of red deer in the north-eastern Alpine range since the pre-Last Glacial Maximum (LGM) period (>25,000 years cal BP) [21]. In this period red deer occupied entire Europe from the Mediterranean peninsulas to the edge of North European Plain and from Ireland in the West to the Black Sea in the East. During the subsequent climatic phase of maximum cold (*c* 25,000–18,000 years cal BP) red deer survived in refugia in the south of Europe, mainly in the Iberian, Italian and Balkans peninsulas. In this period the presence of red deer in Italy is demonstrated in radiometric dated deposits from Arene Candide Cave (Savona) [22], [23], Grotta Paglicci in Gargano (Foggia) [24] and Grotta della Paina in the Colli Berici (Vicenza) [25]. At the end of such an extensive glaciation the red deer populations expanded more or less across all Europe [26].

The abundance of artistic representations of red deer among the archaeological sites in the central and eastern Alps testifies the special relationship between humans and red deer since prehistoric times. In particular, the rock engravings of Val Camonica (Lombardy) depicting scenes of red deer hunting constitute an extraordinary documentation of the central role of red deer among the prehistoric populations in the Alpine arc [21].

Until the end of the 16^th^ century, reed deer was widespread all over the Italian peninsula and Sardinia. Starting from 17th century, however, deforestation, human population growth and hunting led to a gradual extinction of this species from both Alpine and Appennine ranges [27], [28]. At the end of the 19th century the only remnant populations were in the Mesola Wood, in the Po delta area (Emilia Romagna), and in Sardinia.

Currently, population inhabiting the central and eastern Alps derive from spontaneous recolonization from neighboring countries while populations from western Alps and Apennines are the results of reintroduction [28].

The age of the Alpine red deer analyzed in this study (approximately 5,100–5350 years old) offers a unique opportunity to characterize the haplotype of a red deer inhabiting the Alps before any reintroduction or translocation took place.

Our phylogenetic analyses place the ancient Alpine red deer haplotype within the western European mitochondrial lineage (haplogroup A). In addition, the network of the control region haplotypes for the European red deer (Figure 4) shows that the ancient Alpine haplotype is closely related to the haplotypes found in ancient Irish and Norwegian specimens. These findings suggest a common evolutionary history of the Alpine Copper Age red deer and the populations from northern parts of Europe, western and large part of central Europe.

On the other hand, the haplotypes detected in the current populations from the Italian Alps (Friuli, Piedmont) derive from the eastern lineage belonging to haplogroup C. Given the records that document natural recolonization, mainly in central and eastern Alps, and reintroductions, principally in western Alps [28], this data may not surprise if the haplotypes involved in these movements belonged to haplogroup C.

The phylogenetic analyses also show that the haplotype of the Alpine Copper Age red deer differs significantly from the haplotypes of the Sardinian *Cervus elaphus corsicanus* (haplogroup B) and *Cervus elaphus* from Mesola Wood (haplotype AD3).

*C. e. corsicanus* inhabits the Tyrrenian islands of Corsica and Sardinia. The picture of the phylogenetic relationships of *C. e. corsicanus* is still unclear [29], [10]. Nuclear genome analysis based on microsatellites markers on red deer populations from Sardinia, mainland Italy (Val di Susa in Piedmont, and Tarvisio in Friuli-Venezia Giulia), Spain and Bulgaria showed a close relationship between the Sardinian red deer and the red deer from the eastern Italian Alps of Tarvisio. By contrast, the sequencing of the mtDNA control region gave the evidence for a close affinity to the Spanish population and a high genetic divergence with the Tarvisio population [30].

According to the mitochondrial data above reported, our phylogenetic analyses based on *cytb* and mtDNA control region sequences show a high genetic distance between the 5,350–5,100 years old Alpine red deer and the *C.e. corsicanus* from Sardinia/Corsica.

Considering the *Cervus elaphus* from Mesola Wood, previous studies failed to determine if the Mesola haplotype (AD3) belongs to haplogroup A or C or rather represents a remnant of an extinct Italian refugial lineage [11]. Our analyses, unfortunately do not resolve the question showing AD3 branching out at the root of the haplogroup C.

Over the time two hypothesis have been formulated to explain the genetic diversity of the Mesola red deer: the first supports the possibility that Italian peninsula operated as western (haplogroup A) glacial refugium; the second asserts that the genetic diversity of the Mesola red deer is the result of migrations from Iberian glacial refugium (haplogroup A) and consequent prolonged isolation [11]. Given that Mesola haplotype (AD3) is the remnant of an original Italian gene pool and that among all haplotypes of group A is the closest to haplogroup C, Sommer et al., [26] argued that there may have been Italian C haplotypes in the past.

Our analyses indicate the presence of red deer belonging to haplogroup A in the Alpine arc 5,350–5,100 years BP and we can suggest that a post-glacial colonization of type A red deer occurred in the eastern Alps. Moreover we can reasonably hypothesize that the Alpine Copper Age red deer and the Mesola deer have had a common origin from a population of the central Europe genetically affiliated to the western mtDNA lineage. Afterwards, the fragmentation of this ancestral population led to the genetic diversification of the haplotype of Mesola due to prolonged isolation.

Clearly, more molecular studies on archaeological and modern samples of red deer are needed to obtain a better understanding of the phylogenetic relationships of the Copper Age red deer with the current Alpine populations and native red deer of Italy.

## Supporting Information

Figure S1 **Maximum likelihood tree of European cytochrome b haplotypes.** The evolutionary history was inferred by using the Maximum Likelihood method based on the Tamura-Nei model [20]. The tree is drawn to scale, with branch lengths measured in the number of substitutions per site. Numbers on branches indicate bootstrap support (1,000 replicates).
(TIF)

Figure S2 **Maximum likelihood tree of European mtDNA control region haplotypes.** The evolutionary history was inferred by using the Maximum Likelihood method based on the Tamura-Nei model [20]. The tree is drawn to scale, with branch lengths measured in the number of substitutions per site. Numbers on branches indicate bootstrap support (1,000 replicates).
(TIF)

Table S1 **Primer systems utilized with the corresponding product length and annealing temperature.**
(DOC)

Table S2 **List of the species used to construct the** ***Cervus elaphus*** **maximum likehood worldwide tree (** **Figure 1** **) of cytochrome b sequences.**
(DOC)

Table S3 **List of the species used to construct the** ***Cervus elaphus*** **maximum likehood worldwide tree (** **Figure 2** **) of mtDNA control region sequences.**
(DOC)

Table S4 **List of the European cytochrome b haplotypes used to construct the haplotype network (** **Figure 3** **) and the maximum likehood tree (Figure S1).**
(DOC)

Table S5 **List of the European mtDNA control region haplotypes used to construct the haplotype network (** **Figure 4** **) and the maximum likehood tree (Figure S2).**
(DOC)

Table S6 **Nucleotide misincorporations within each mtDNA clonal group.**
(DOC)
