# Supplementary figures and images for: Positioning the Red Deer (Cervus elaphus) Hunted by the Tyrolean Iceman into a Mitochondrial DNA Phylogeny
Source: PLoS One. 2014 Jul 2;9(7):e100136. doi: 10.1371/journal.pone.0100136 (PMC4079593; doi:10.1371/journal.pone.0100136)

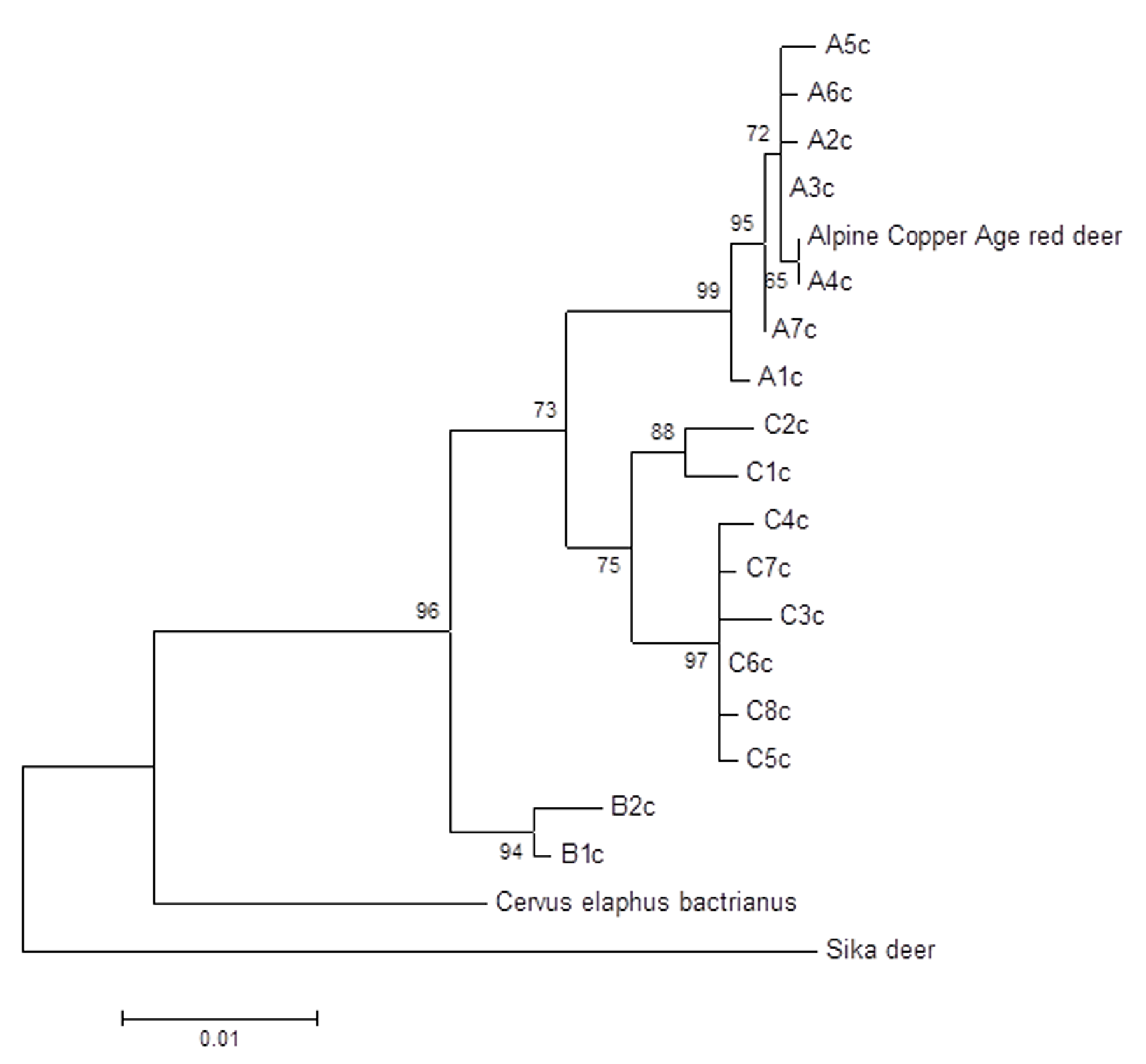

Supplement: Figure S1 — Maximum likelihood tree of European cytochrome b haplotypes. The evolutionary history was inferred by using the Maximum Likelihood method based on the Tamura-Nei model [20]. The tree is drawn to scale, with branch lengths measured in the number of substitutions per site. Numbers on branches indicate bootstrap support (1,000 replicates). (TIF) [file pone.0100136.s001.tif]
