# Supplementary material for: Positioning the Red Deer (Cervus elaphus) Hunted by the Tyrolean Iceman into a Mitochondrial DNA Phylogeny
Source: PLoS One. 2014 Jul 2;9(7):e100136. doi: 10.1371/journal.pone.0100136 (PMC4079593; doi:10.1371/journal.pone.0100136)
Supplement: Table S1 — Primer systems utilized with the corresponding product length and annealing temperature. (DOC) [file pone.0100136.s003.doc]

**Table S1. Primer systems utilized with the corresponding product length and annealing temperature**

| **Forvard** | **Sequence (5'→ 3')** | **Reverse** | **Sequence (5'→ 3')** | **Length (bp)** | **AT (°C)** |
| --- | --- | --- | --- | --- | --- |
| Cervus elaphus L14146 | GAAAAACCATCGTTGTCATTCA | Cervus elaphus H14297 | GCTAGGAATAGGCCTGTGAG | 193 | 57 |
| Cervus elaphus L14225 | CAACGCATTTATTGACCTCC | Cervus elaphus H14353 | CGACAGATATGGGTGACAGAG | 183 | 57 |
| Cervus elaphus L14316 | TCACAGGCCTATTCCTAGC | Cervus elaphus H14411 | AATATTGATGCCCCGTTTG | 133 | 56 |
| Cervus elaphus L14375 | GTCACCCATATCTGTCGAG | Cervus elaphus H14526 | GAATGCTGTGGCTATAACTG | 191 | 53 |
| Cervus elaphus L14439 | GCATCAATATTTTTCATCTG | Cervus elaphus H14568 | TCCTCAGAATGATATTTGTC | 169 | 49 |
| Cervus elaphus L14541 | ATTTACAGTTATAGCCACAGC | Cervus elaphus H14638 | CAGATCCATTCGACTAGG | 134 | 50 |
| Cervus elaphus L14589 | ACAAATATCATTCTGAGGAGC | Cervus elaphus H14718 | AGTGCTGCGATGATAAATG | 167 | 53 |
| Cervus elaphus L14699 | AACCCTAACCCGATTTTTCG | Cervus elaphus H14795 | ATTTTGTCTGCGTCTGATGG | 134 | 58 |
| Cervus elaphus L14773 | CTTACTCTTCCTTCACGAAACAGG | Cervus elaphus H14898 | TCTCCAAGTAGGTCTGGTGC | 172 | 58 |
| Cervus elaphus L14853 | ACCATTAAAGATATCTTAGG | Cervus elaphus H14976 | GTATGCAAATAGGAAATATC | 161 | 45 |
| Cervus elaphus L14952 | CCAGCAAACCCACTCAACAC | Cervus elaphus H15098 | GGTCGGAATATCATGCTGCG | 224 | 57 |
| Cervus elaphus L15087 | TCATGCCTCTTCTTCACAC | Cervus elaphus H15212 | AAGACAGATGCTAGTTGTCC | 162 | 52 |
| Cervus elaphus L15176 | TAACACTTACATGAATCG | Cervus elaphus H15319 | CAAGACCAGTGTATTGAG | 179 | 54 |
| Cervus elaphus bl L15607 | CTTATGCGCTTATAGTACATAG | Cervus elaphus bl H15660 | GTACATGCTTATATGCATGG | 171 | 49 |
| Cervus elaphus L15308 | CCTCCTAAAATGAAGATAAGTC | Cervus elaphus H15435 | CTATATTAATAAGCATCAGGG | 126 | 48 |
| Cervus elaphus L15396 | CAAGGAAGAAGCCATAGCCCC | Cervus elaphus H15512 | GGTGTTGAGTGGAAAGCTGTATTG | 160 | 61 |
| Cervus elaphus L15456 | CCCTGATGCTTATTAATATAG | Cervus elaphus H15587 | CTATGTACTATAAGCGCATAAG | 174 | 47 |
| Cervus elaphus L15536 | GCTTTCCACTCAACACCC | Cervus elaphus H15644 | GCATGGGGCATATAATATAATG | 147 | 55 |
| Cervus elaphus L15616 | GCGCTTATAGTACATAGAATTAATG | Cervus elaphus H15780 | ATGGTGATCAAGCTCGTG | 205 | 53 |
| Cervus elaphus L15739 | ATCGTACATAGCGCATTAAGTC | Cervus elaphus H15851 | CTACCCCCACAGTTCATG | 149 | 54 |
| Cervus elaphus L15812 | ACCATGCCGCGTGAAACCAG | Cervus elaphus H15929 | GTCTTATATTGCAAGGAGTGGGCG | 159 | 54 |
| Cervus elaphus L15909 | GACATCTGGTTCTTTTTTCAG | Cervus elaphus H16074 | GCTACAATTCATGCTCCG | 202 | 53 |
| Cervus elaphus L16049 | GGGATGCTTGGACTCAGC | Cervus elaphus H16181 | GGGAAAATAGATCTTAGGGTTG | 172 | 53 |
| Cervus elaphus L16155 | ATGGCAGTCAATGGTCAC | Cervus elaphus H16332 | ATTAGGGAAAAATTGGCG | 211 | 54 |
| Cervus elaphus L16309 | AATACTCAAATCAGCACTC | Cervus elaphus H110 | GATGCTTGCATGTGTAAG | 193 | 48 |
