# Supplementary material for: Positioning the Red Deer (Cervus elaphus) Hunted by the Tyrolean Iceman into a Mitochondrial DNA Phylogeny
Source: PLoS One. 2014 Jul 2;9(7):e100136. doi: 10.1371/journal.pone.0100136 (PMC4079593; doi:10.1371/journal.pone.0100136)
Supplement: Table S2 — List of the species used to construct the Cervus elaphus maximum likehood worldwide tree ( Figure 1 ) of cytochrome b sequences. (DOC) [file pone.0100136.s004.doc]

**Table S2. List of the species used to construct the *Cervus elaphus* maximum likehood worldwide tree (Figure 1) of cytochrome b sequences.**

| **Species name** | **Country** | **Accession Number** | **Reference** |
| --- | --- | --- | --- |
| Alpine Copper Age *red deer* | Eastern Alps |  | This work |
| *C.e.canadensis 1* | USA | AB021096 | [1] |
| *C.e.kansuensis 1* | China | AB021098 | [1] |
| *C.e.scoticus* | Scotland | AB021099 | [1] |
| *C.e.hippelaphus* | Turkey | AY118197 | [2] |
| *C.e.hippelaphus* | Austria | AY044857 | [2] |
| *C.e.wallichi* | China | AY044861 | [2] |
| *C.e.* | New Zealand | NC007704 | [3] |
| *C.e.hippelaphus* | Bulgaria | AF423195 | [2] |
| *C.e.xanthopygus 1* | Russia | AF423197 | [2] |
| *C.e.sibericus 1* | China | AF423199 | [2] |
| *C.e.barbarus* | Tunisia | AY070222 | [2] |
| *C.e.xanthopygus 2* | Russia | AY070224 | [2] |
| *C.e.atlanticus 1* | Norway | AY070226 | [2] |
| *C.e.maral* | Iran | AF489280 | [2] |
| *C.e.maral* | Turkey | AY118199 | [2] |
| *C.e.* | China | AY044856 | [2] |
| *C.e. hippelaphus* | Germany | AY044858 | [2] |
| *C.e.hippelaphus* | Poland | AY044860 | [2] |
| *C.e.sibericus 2* | China | AY044862 | [2] |
| *C.e.hippelaphus* | Germany | AF423196 | [2] |
| *C.e.canadensis 2* | USA | AF423198 | [2] |
| *C.e.atlanticus 2* | Norway | AY070221 | [2] |
| *C.e.kansuensis 2* | China | AY070223 | [2] |
| *C.e.hippelaphus* | Yugoslavia | AY070225 | [2] |
| *C.e.hippelaphus* | Hungary | AF489279 | [2] |
| *C.e.hispanicus* | Spain | AF489281 | [2] |
| *C.e.barbarus* | Germany | AY118198 | [2] |
| *C.e.bactrianus* | China | AY142327 | [2] |
| *C.e.* | Mongolia | AY244490 | [2] |
| *C.e.yarkandensis* | China | AY142326 | [2] |
| *C.e.hippelaphus* | Ukraine | AY148966 | [2] |
| *C.e.corsicanus* | Italy-Sardinia | AY244489 | [2] |
| *C.e.hippelaphus* | France | AY244491 | [2] |
| *C.e.macneilli* | China | AY035875 | [2] |
| *C.e.songaricus* | China | AY035871 | [2] |
| *Cervus nippon centralis* | Japon | AB021094 | [2] |
| *Cervus nippon yesoensis* | Japon | AB021095 | [2] |
| *Cervus nippon mageshimae* | Japon | AB021092 | [2] |
| *Cervus nippon nippon* | Japon | AB021093 | [2] |
| *Cervus albirostris 1* | China | AY044863 | [2] |
| *Cervus albirostris 2* | China | AF423202 | [2] |
| *Cervus timorensis macassanicus* | Indonesia | AB423200 | [2] |
| *Cervus unicolor cambojensis* | China | AF423201 | [2] |
| *Dama dama* |  | NC020700 | [4] |

**References**

1. Kuwayama R, Ozawa T (2000) Phylogenetic relationships among european red deer, wapiti, and sika deer inferred from mitochondrial DNA sequences. Mol Phylogenet Evol 15 (1): 115-123.

2. Ludt CJ, Schroeder W, Rottmann O, Kuehn R (2004) Mitochondrial DNA phylogeography of red deer (Cervus elaphus). Mol Phylogenet Evol 31: 1064-1083.

3. Wada K, Okumura K, Nishibori M, Kikkawa Y, Yokohama M (2010) The complete mitochondrial genome of the domestic red deer (Cervus elaphus) of New Zealand and its phylogenic position within the family Cervidae. Anim Sci J 81: 551-557.

4. Hassanin A, Delsuc F, Ropiquet A, Hammer C, Jansen van Vuuren B, et al., (2012) Pattern and timing of diversification of Cetartiodactyla (Mammalia, Laurasiatheria), as revealed by a comprehensive analysis of mitochondrial genomes. CR Biol 335 (1): 32-50.
