# Supplementary material for: Positioning the Red Deer (Cervus elaphus) Hunted by the Tyrolean Iceman into a Mitochondrial DNA Phylogeny
Source: PLoS One. 2014 Jul 2;9(7):e100136. doi: 10.1371/journal.pone.0100136 (PMC4079593; doi:10.1371/journal.pone.0100136)
Supplement: Table S3 — List of the species used to construct the Cervus elaphus maximum likehood worldwide tree ( Figure 2 ) of mtDNA control region sequences. (DOC) [file pone.0100136.s005.doc]

**Table S3. List of the species used to construct the *Cervus elaphus* maximum likehood worldwide tree (Figure 2) of mtDNA control region sequences.**

| **Species name** | **Country** | **Accession**  **Number** | **Reference** |
| --- | --- | --- | --- |
| Alpine Copper Age red deer | Eastern Alps |  | This work |
| *C.e. 1* | Scotland | DQ386110 | [1] |
| *C.e. 2* | Scotland | DQ386109 | [1] |
| *C.e. 3* | Scotland | DQ386107 | [1] |
| *C.e. 4* | Scotland | DQ386106 | [1] |
| *C.e. 5* | Scotland | DQ386108 | [1] |
| *C.e.* | New Zealand | NC007704 | [2] |
| *C.e. hippelaphus* | North Italy | AF291887 | [3] |
| *C.e. hippelaphus* | South Italy | AF291886 | [3] |
| *C.e. barbarus 1* | Tunisia | AF296807 | [4] |
| *C.e. barbarus 2* | Tunisia | AF296808 | [4] |
| *C.e hispanicus* | Spain | AF291889 | [3] |
| *C.e atlanticus* | Norway | AF291888 | [3] |
| *C.e. macreilli 1* | China | AF296809 | [4] |
| *C.e. macreilli 2* | China | AF296810 | [4] |
| *C.e. macreilli 3* | China | AF296811 | [4] |
| *C.e. macreilli 4* | China | AF296812 | [4] |
| *C.e. macreilli 5* | China | AF296813 | [4] |
| *C.e. macreilli 6* | China | AF296814 | [4] |
| *C.e. kansuensis* | China | AF296819 | [4] |
| *C.e. nelsoni 1* | America | AF016964 | [4] |
| *C.e. nelsoni 2* | America | AF016979 | [4] |
| *C.e. manitobensis* | America | AF016957 | [4] |
| *C.e. nannodes* | America | AF016976 | [4] |
| *C. e. nelsoni 3* | America | AF291882 | [3] |
| *C. e. nelsoni 4* | America | AF016980 | [4] |
| *C. e. roosevelti* | America | AF016970 | [4] |
| *C. e. sibericus 1* | Asia | AF058371 | [4] |
| *C. e. sibericus 2* | Asia | AF058370 | [4] |
| *C. e. sibericus 3* | Asia | AF058369 | [4] |
| *C. e. xanthopygus* | Asia | AF296817 | [4] |
| *C. e. alashanicus* | Asia | AF296818 | [4] |
| *C. nippon 1* | Japan | AF016974 | [4] |
| *C. nippon 2* | Japan | AF016975 | [4] |
| *C. e. bactrianus 1* | China | AF296822 | [4] |
| *C. e. bactrianus 2* | China | AF296821 | [4] |
| *C. e. bactrianus 3* | China | AF296820 | [4] |
| *Dama dama* |  | NC020700 | [5] |

**References**

1.Nussey DH, Pemberton J, Donald A, Kruuk LE (2006) Genetic consequences of human management in an introduced island population of red deer (Cervus elaphus). Heredity 97 (1): 56-65.

2.Wada K, Okumura K, Nishibori M, Kikkawa Y, Yokohama M (2010) The complete mitochondrial genome of the domestic red deer (Cervus elaphus) of New Zealand and its phylogenic position within the family Cervidae. Anim Sci J 81: 551-557.

3.Randi E, Mucci N, Claro-Hergueta F, Bonnet A, Douzery EJP (2001) A mitochondrial DNA control region phylogeny of the Cervinae: speciation in Cervus and implications for conservation. Anim Conserv 4: 1-11.

4. Polziehn RO, Strobeck C (2002) A phylogenetic comparison of red deer and wapiti using mitochondrial DNA. Mol Phylogenet Evol 22 (3): 342-356.

5. Hassanin A, Delsuc F, Ropiquet A, Hammer C, Jansen van Vuuren B, et al., (2012) Pattern and timing of diversification of Cetartiodactyla (Mammalia, Laurasiatheria), as revealed by a comprehensive analysis of mitochondrial genomes. CR Biol 335 (1): 32-50.
