# Supplementary material for: Positioning the Red Deer (Cervus elaphus) Hunted by the Tyrolean Iceman into a Mitochondrial DNA Phylogeny
Source: PLoS One. 2014 Jul 2;9(7):e100136. doi: 10.1371/journal.pone.0100136 (PMC4079593; doi:10.1371/journal.pone.0100136)
Supplement: Table S4 — List of the European cytochrome b haplotypes used to construct the haplotype network ( Figure 3 ) and the maximum likehood tree (Figure S1). (DOC) [file pone.0100136.s006.doc]

**Table S4. List of the European cytochrome b haplotypes used to construct the haplotype network (Figure 3) and the maximum likehood tree (Figure S1).**

| **Haplotype** | **Country** | **Accession number** | **Reference** |
| --- | --- | --- | --- |
| Alpine Copper Age *Cervus elaphus* | Eastern Alps |  | This study |
| A2c | Spain | DQ524849 | [1] |
| C7c | The Czech Republic | DQ524848 | [1] |
| C8c | Friuli, Piedmont,  The Czech Republic | DQ524847 | [1] |
| A4c | France | AY244491 | [1] |
| B2c | Sardinia | AY244489 | [1] |
| A1c | Ukraine | AY148966 | [1] |
| C2c | Turkey | AY118199 | [1] |
| A5c | Spain | AF489281 | [1] |
| C1c | Iran | AF489280 | [2] |
| C4c | Bulgaria | AF423195 | [1] |
| A7c | Poland | AY044860 | [1] |
| A6c | Germany, Piedmont | AY044858 | [1] |
| C3c | Austria | AY044857 | [2] |
| C5c | Turkey, Hungary, The Czech Republic | AY118197 | [1] |
| B1c | Tunisia | AY070222 | [1] |
| A3c | Sweden, Norway, France, Spain, Scotland | AB021099 | [1] |
| C6c | Romania, The Czech Republic, Former Yugoslavia | AY070225 | [2] |
| *Cervus elaphus bactrianus* |  | AY142327 | [2] |
| Sika deer (*Cervus nippon*) |  | AY035876 | [2] |

**References**

1**.** Skog A, Zachos FE, Rueness EK, Feulner PGD, Mysterud A, et al. (2009) Phylogeography of red deer (Cervus elaphus) in Europe. J Biogeogr 36: 66-77.

2. Ludt CJ, Schroeder W, Rottmann O, Kuehn R (2004) Mitochondrial DNA phylogeography of red deer (Cervus elaphus). Mol Phylogenet Evol 31: 1064-1083.
