# Supplementary material for: Positioning the Red Deer (Cervus elaphus) Hunted by the Tyrolean Iceman into a Mitochondrial DNA Phylogeny
Source: PLoS One. 2014 Jul 2;9(7):e100136. doi: 10.1371/journal.pone.0100136 (PMC4079593; doi:10.1371/journal.pone.0100136)
Supplement: Table S5 — List of the European mtDNA control region haplotypes used to construct the haplotype network ( Figure 4 ) and the maximum likehood tree (Figure S2). (DOC) [file pone.0100136.s007.doc]

**Table S5. List of the European mtDNA control region haplotypes used to construct the haplotype network (Figure 4) and the maximum likehood tree (Figure S2).**

| **Haplotype** | **Country** | **Accession number** | **Reference** |
| --- | --- | --- | --- |
| Alpine Copper Age *Cervus elaphus* | Eastern Alps |  | This Study |
| BC | Sardinia, Spain | DQ520256 | [1] |
| BD | Sardinia | DQ520255 | [1] |
| BF | Sardinia | DQ520254 | [1] |
| BE | Sardinia | DQ520253 | [1] |
| BA | Sardinia | DQ520252 | [1] |
| BB | Sardinia | DQ520251 | [1] |
| CA6 | Romania | DQ520250 | [1] |
| CA2 | Piedmont | DQ520249 | [1] |
| CA1 | Bulgaria, Romania, Serbia, Piedmont | DQ520248 | [1] |
| CA5 | Serbia, Hungary | DQ520247 | [1] |
| CA3 | Romania, The Czech Republic | DQ520246 | [1] |
| CA4 | Romania, Friuli | DQ520245 | [1] |
| CB1 | Friuli, Piedmont | DQ520244 | [1] |
| CB2 | Friuli | DQ520237 | [1] |
| CB3 | Piedmont | DQ520236 | [1] |
| CB5 | Friuli, Piemonte, Romania, The Czech Republic | DQ520235 | [1] |
| CB4 | Piedmont | DQ520234 | [1] |
| CD5 | Bulgaria | DQ520243 | [1] |
| CD3 | Hungary | DQ520242 | [1] |
| CD2 | Romania | DQ520241 | [1] |
| BG | Algeria | AF296807 | [2] |
| CD4 | Bulgaria, Romania | DQ520240 | [1] |
| AD7 | Romania | DQ520205 | [1] |
| CD1 | Friuli, Piedmont, Romania, Hungary, The Czech Republic | DQ520238 | [1] |
| CD6 | Romania | DQ520239 | [1] |
| AD3 | Mesola (Emilia Romagna) | DQ520224 | [1] |
| AB3 | Germany | DQ520226 | [1] |
| AC5 | Germany | DQ520209 | [1] |
| AC6 | Germany | DQ520214 | [1] |
| AC7 | Germany | DQ520210 | [1] |
| AC2 | Sweden | DQ520218 | [1] |
| AC1 | Norway | DQ520221 | [1] |
| AC3 | Norway | DQ520220 | [1] |
| AA7 | Norway | DQ520215 | [1] |
| AC4 | Norway | DQ520223 | [1] |
| AA1 | Norway,Spain | DQ520219 | [1] |
| AA9 | France | DQ520200 | [1] |
| AB2 | Piedmont | DQ520225 | [1] |
| AA6 | Sardinia | DQ520228 | [1] |
| AD1 | France | DQ520202 | [1] |
| AA3 | France | DQ520203 | [1] |
| AD2 | France | DQ520201 | [1] |
| AA8 | France | DQ520222 | [1] |
| AA4 | Spain | DQ520229 | [1] |
| AA5 | Spain | DQ520227 | [1] |
| AA10 | Spain | DQ520207 | [1] |
| AA2 | Spain | DQ520204 | [1] |
| AB1 | Spain | DQ520206 | [1] |
| AB4 | Spain | DQ520230 | [1] |
| AB6 | Spain | DQ520231 | [1] |
| AB5 | Spain | DQ520232 | [1] |
| AB7 | Spain | DQ520233 | [1] |
| AD4 | Scotland | DQ520216 | [1] |
| AD5 | Scotland | DQ520213 | [1] |
| AD8 | Scotland | DQ520211 | [1] |
| AD6 | Scotland | DQ520212 | [1] |
| AD9 | Scotland | DQ520208 | [1] |
| AD10 | Scotland | DQ520217 | [1] |
| IRE1 | Ireland | JQ599359 | [3] |
| IRE2 | Ireland | JQ599360 | [3] |
| IRE3 | Ireland | JQ599361 | [3] |
| IRE4 | Ireland | JQ599362 | [3] |
| IRE5 | Ireland | JQ599363 | [3] |
| IRE6 | Ireland | JQ599364 | [3] |
| IRE7 | Ireland | JQ599365 | [3] |
| IRE8 | Ireland | JQ599366 | [3] |
| IRE9 | Ireland | JQ599367 | [3] |
| IRE10 | Ireland | JQ599368 | [3] |
| IRE11 | Ireland | JQ599369 | [3] |
| IRE12 | Ireland | JQ599370 | [3] |
| IRE13 | Ireland | JQ599371 | [3] |
| IRE14 | Ireland | JQ599372 | [3] |
| NO1 | Norway | JX861260 | [4] |
| NO2 | Norway | JX861261 | [4] |
| NO3 | Norway | JX861262 | [4] |
| NO4 | Norway | JX861263 | [4] |
| NO5 | Norway | JX861264 | [4] |
| NO6 | Norway | JX861265 | [4] |
| NO7 | Norway | JX861266 | [4] |
| NO8 | Norway | JX861267 | [4] |
| NO9 | Norway | JX861268 | [4] |
| NO10 | Norway | JX861269 | [4] |
| *Cervus elaphus bactrianus* |  | AF296823 | [5] |
| Sika deer (*Cervus nippon*) |  | JF893526 | [6] |

**References**

1. Skog A, Zachos FE, Rueness EK, Feulner PGD, Mysterud A, et al. (2009) Phylogeography of red deer (Cervus elaphus) in Europe. J Biogeogr 36: 66-77.

2. Ludt CJ, Schroeder W, Rottmann O, Kuehn R (2004) Mitochondrial DNA phylogeography of red deer (Cervus elaphus). Mol Phylogenet Evol 31: 1064-1083.

3. Carden RF, McDevitt AD, Zachos FE, Woodman PC, O'Toole P, et al. (2012) Phylogeographic, ancient DNA, fossil and morphometric analyses reveal ancient and modern introductions of a alrge mammal: the complex case of red deer (cervus elaphus) in Ireland. Quaternary Sci Rev 42: 74-84.

4. Rosvold J, Røed KH, Huthammer AK, Andersen R, Stenøien HK (2012) Reconstructing the history of a fragmented and heavily exploited red deer population using ancient and contemporary DNA. BMC Evol Biol 12: 191.

5. Polziehn RO, Strobeck C (2002) A phylogenetic comparison of red deer and wapiti using mitochondrial DNA. Mol Phylogenet Evol 22 (3): 342-356.

6. Barancekova M, Krojerova-Prokesova J, Voloshina IV, Myslenkov AI, Kawata Y, et al., (2012) The origin and genetic variability of the Czech sika deer population. Ecol Res 27 (6): 991-1003.
