# Supplementary material for: Positioning the Red Deer (Cervus elaphus) Hunted by the Tyrolean Iceman into a Mitochondrial DNA Phylogeny
Source: PLoS One. 2014 Jul 2;9(7):e100136. doi: 10.1371/journal.pone.0100136 (PMC4079593; doi:10.1371/journal.pone.0100136)
Supplement: Table S6 — Nucleotide misincorporations within each mtDNA clonal group. (DOC) [file pone.0100136.s008.doc]

**Table S6.** Nucleotide misincorporations within each mtDNA clonal group

| **Clonal group** | **A+T/G+C Ratio** | **A→G T→C Type 1** | **C→T G→A Type 2** | **A→C T→G** | **A→T T→A** | **C→A G→T** | **C→G G→C** |
| --- | --- | --- | --- | --- | --- | --- | --- |
|
| Cervus elaphus L14146/Cervus elaphus H14297 | 1,7 | 894 | 2434,4 | 505 | 884 | 861,9 | 537,2 |
| Cervus elaphus L14225/Cervus elaphus H14353 | 1,46 | 412 | 1103,76 | 346 | 118 | 477,42 | 557,72 |
| Cervus elaphus L14316/Cervus elaphus H14411 | 1,71 | 247 | 275,31 | 98 | 154 | 148,77 | 169,29 |
| Cervus elaphus L14375/Cervus elaphus H14526 | 1,64 | 745 | 1126,68 | 235 | 445 | 613,36 | 155,8 |
| Cervus elaphus L14439/Cervus elaphus H14568 | 1,38 | 583 | 1106,76 | 187 | 292 | 276 | 248,4 |
| Cervus elaphus L14541/Cervus elaphus H14638 | 1,42 | 541 | 667,4 | 174 | 185 | 198,8 | 306,72 |
| Cervus elaphus L14589/Cervus elaphus H14718 | 1,3 | 1559 | 1870,7 | 222 | 451 | 683,8 | 174,2 |
| Cervus elaphus L14699/Cervus elaphus H14795 | 1,46 | 1161 | 2074,66 | 374 | 227 | 854,1 | 121,18 |
| Cervus elaphus L14773/Cervus elaphus H14898 | 1,023 | 439 | 289,509 | 184 | 170 | 173,91 | 99,231 |
| Cervus elaphus L14853/Cervus elaphus H14976 | 1,53 | 358 | 908,82 | 969 | 235 | 1447,4 | 117,81 |
| Cervus elaphus L14952/Cervus elaphus H15098 | 1,43 | 505 | 1202,63 | 844 | 169 | 1637,4 | 204,49 |
| Cervus elaphus L15087/Cervus elaphus H15212 | 1,5 | 642 | 2026,5 | 254 | 363 | 162 | 73,5 |
| Cervus elaphus L15176/Cervus elaphus H15319 | 1,7 | 423 | 1026,8 | 227 | 354 | 100,3 | 71,4 |
| Cervus elaphus bl L15607/Cervus elaphus bl H15660 | 2,79 | 725 | 1905,57 | 109 | 94 | 315,27 | 270,63 |
| Cervus elaphus L15308/Cervus elaphus H15435 | 1,44 | 283 | 381,6 | 343 | 352 | 424,8 | 704,16 |
| Cervus elaphus L15396/Cervus elaphus H15512 | 3 | 1863 | 585 | 961 | 1080 | 498 | 534 |
| Cervus elaphus L15456/Cervus elaphus H15587 | 2,64 | 2899 | 5045,04 | 1824 | 2709 | 2154,2 | 630,96 |
| Cervus elaphus L15536/Cervus elaphus H15644 | 2,48 | 1980 | 3132,24 | 1044 | 1906 | 2013,8 | 508,4 |
| Cervus elaphus L15616/Cervus elaphus H15780 | 1,88 | 1765 | 3500,56 | 750 | 1534 | 962,56 | 580,92 |
| Cervus elaphus L15739/Cervus elaphus H15851 | 0,72 | 985,32 | 1918 | 251,16 | 202,86 | 377 | 433 |
| Cervus elaphus L15812/Cervus elaphus H15929 | 0,98 | 2717,424 | 3886 | 289,85 | 364,09 | 1029 | 1724 |
| Cervus elaphus L15909/Cervus elaphus H16074 | 1,11 | 331 | 888 | 259 | 238 | 751,47 | 1401,9 |
| Cervus elaphus L16049/Cervus elaphus H16181 | 1,03 | 144 | 371,83 | 37 | 22 | 93,73 | 40,17 |
| Cervus elaphus L16155/Cervus elaphus H16332 | 1,91 | 141 | 242,57 | 11 | 30 | 34,38 | 22,92 |
| Cervus elaphus L16309/Cervus elaphus H110 | 1,63 | 31 | 74,98 | 5 | 3 | 22,82 | 21,19 |
